# Retrospective longitudinal analysis of the effects of postnatal weight gain on the timing and *tempo* of puberty and menarche in a cohort of Italian girls

**DOI:** 10.1186/s13052-022-01222-9

**Published:** 2022-02-03

**Authors:** Vittorio Ferrari, Simona Stefanucci, Marta Ferrari, Daniele Ciofi, Stefano Stagi, on the behalf of the Tuscany Menarche Study Group, Antonio Milanesi, Rossana Cecchi, Rosalba Fiore, Monica Pierattelli, Angela Maria Pittari, Antonina Chiccoli, Paolo Becherucci, Anna Cova, Tiziana Guidotti, Elena Balzer, Giovanni Scipione Gaetano Citino, Paolo Bagni, Marzia Guarnieri, Roberto Pecchioli, Lara Ascani, Donatella Matteoni, Patrizia Beacci, Vanda Lelli, Cecilia Breschi, Cristina Fantacci, Anna De Simone, Manuela Gabbrielli

**Affiliations:** Department of Health Sciences, University of Florence, Anna Meyer Children’s University Hospital, viale Pieraccini 24, Florence, Italy

**Keywords:** Children, Postnatal weight gain, Age at thelarche, Age at menarche, Secular trend, Puberty, Onset of Puberty, Overweight, Public health, Epidemiology

## Abstract

**Objective:**

over the last few decades there has been a progressive decline in the average age of onset of pubertal development stages in both sexes. The increase in the prevalence of childhood obesity seems to play an important role in this phenomenon.

**Design:**

we undertook a retrospective, longitudinal evaluation of the average age of thelarche and menarche to evaluate the relationship between BMI and weight change during the first years of life and the timing and *tempo* of puberty.

**Methods:**

we evaluated data for 577 Italian girls born between 1995 and 2003. We collected the main auxological and clinical parameters, including age at B2 and at menarche, BMI SDS at B2 and menarche, gestational age and birth weight and Z-score change from birth weight (BW) to BMI at B2 and menarche.

**Results:**

the mean age of B2 was 10.06 ± 1.03 years and the mean age of menarche was 12.08 ± 1.02 years. Age at B2 and menarche were inversely correlated with BMI SDS (*p* < 0.0001). Both age at menarche and at thelarche have an inverse relationship with the Z-score change from birth weight and BMI at menarche and thelarche respectively (*p* < 0.0001).

**Conclusions:**

our data confirm a significant relationship between BMI and age of B2 and menarche. We observed a clear relationship among weight change during the first years of life, age at thelarche and menarche and the duration of puberty, demonstrating the importance of weight and weight gain in determining the timing and *tempo* of pubertal changes and growth.

## Introduction

The average age of pubertal development has decreased all over the world, particularly in females [1]. The reasons for this phenomenon include changes in nutritional status, an increase in the prevalence of childhood obesity and increasingly frequent exposure to endocrine-disruptors [1, 2]. Some data carried out during the Sars-CoV2 pandemic underlined the importance of environmental factors in the timing and *tempo* of pubertal development, showing an increase of central precocious and fast puberty (CPP) in girls during and following the first lockdown in Italy from March to May 2020 [3, 4]. Among the factors hypothesized to be related to this phenomenon were nutritional factors related to an increased BMI, an “overuse” of electronic devices and psychological triggers [3].

In girls with a normal BMI, there is significant evidence, although mostly from cross-sectional studies [5–7], indicating that BMI influences the timing of thelarche [2, 5, 6, 8] and menarche [2, 7]. Increased adiposity appears to decrease the age of onset and accelerate the progression of puberty. Studies indicate that a larger gain in BMI during childhood whether in the first 20 months [8], in the first 5 years [9] or between the ages of two and eight years [10] is related to an earlier onset of puberty. There also appears to be a relationship between age of pubertal onset and weight at birth [9, 11].

Insulin resistance and excess hepato-visceral fat may play an important role in determining the appearance of the first signs of pubertal development. In these subjects, the early appearance of pubertal development could be a mechanism to minimise increases in central ectopic fat [12]. According to this theory, the Z-score change from birth weight (BW) to BMI in childhood would be a good marker of the metabolic conditions influencing the timing and *tempo* of puberty in normal girls [12].

In this study we evaluated the relationship between Z-score change from BW to BMI at thelarche and menarche and the timing and *tempo* of puberty in a large cohort of Tuscany girls.

## Patients and methods

We carried out a monocentric, retrospective, longitudinal and observational study. We collected the data of 577 girls born between 1995 and 2003 who had reached menarche by the time of the study. We extrapolated the data from the program 'Infantia 2000', commonly used by family paediatricians in Italy, selecting 22 paediatric clinics in Tuscany (central Region of Italy), on the basis of the auxological skills of the paediatricians and the frequency of follow-up visits.

The data collected from medical records was: weight, height, BMI (and respective standard deviation score (SDS)), age of onset of thelarche and menarche (self-reported by families at the time of the nearest visit), weight and height at birth (and respective SDS), maternal and paternal information, history of gestational diabetes, gestational and delivery information.

To calculate the standard deviation scores of the neonatal data (weight and length at birth) we considered the Italian Neonatal Study [INeS] charts [13]; for the auxological parameters (height, weight and BMI at the time of thelarche and menarche) we used the Italian cross-sectional growth charts of Cacciari et al [14]. Girls with a BMI below the 5^th^ centile were considered as underweight, those above the 85^th^ centile overweight, while all others were defined as normal weight [15].

The stages of pubertal maturation were assessed according to the Tanner and Whitehouse criteria [16]. The age of pubertal onset was defined as the age at durable Tanner B2 stage. The duration of puberty was considered as the period between the onset of thelarche and menarche. Final stature was the height reached by girls at least 2 years after menarche.

The exclusion criteria were: adoption; hormonal treatment for endocrinological disorders; therapies based on glucocorticoids, chemotherapy or GnRH analogues; history of chronic diseases such as celiac disease, anorexia, and history of tumours; girls who were SGA (defined as a weight and/or length less than 2 standard deviation [17]) were also excluded because more prone to earlier pubertal development and menarche, and faster progression of puberty than children born appropriate for gestational age (AGA) [18, 19]. To evaluate the influence of the accumulation of central ectopic fat on the age of pubertal development we used the formula described by de Zegher et al [12, 20].

## Results

The mean age of thelarche was 10.06 ± 1.03 years and the mean age of menarche was 12.08 ± 1.02 years. For girls with a normal BMI, the mean ages of B2 and menarche were 10.15 ± 0.99 years and 12.13 ± 0.95 years, respectively. For girls with a low BMI, B2 occurred on average at 10.62 ± 1.02 years and menarche at 13.20 ± 1.15 years (*p* < 0.0001), significantly later than girls with higher BMIs. Overweight girls reached B2 and menarche significantly earlier, on average at 9.47 ± 0.93 years and 11.27 ± 0.83 years (*p* < 0.0001) respectively (Table 1). The age of thelarche and menarche was inversely related to the BMI SDS (respectively *R* = 0.34 and 0.46; *p* < 0.0001).

**Table 1:** Thelarche and menarche ages in relation to the BMI growth chart centiles^a^

BMI (centiles)	Subjects (%)	Thelarche age (yrs)	Subjects (%)	Menarche age (yrs)
Underweight	32 (5.5)	10.62 ± 1.02	30 (5.2)	13.20 ± 1.15
Normal weight	457 (79.2)	10.15 ± 0.99	478 (82.8)	12.13 ± 0.95
Overweight	88 (15.3)	9.47 ± 0.93	69 (12.0)	11.27 ± 0.83

^a^According the Italian cross-sectional growth charts of Cacciari et al. [14]; Underweight: BMI below the 5th centile; normal weight: BMI between 5 and 85^th^ centile; overweight: BMI above the 85th centile[15]

The mean gestational age of our study group was 39.9 ± 1.6 weeks. We did not observe statistically significant differences in the age of thelarche and menarche in preterm (< 37 weeks) and full term girls. Nor did we observe a statistically significant correlation between age at thelarche and menarche and gestational age and length at birth.

**Fig. 1:**
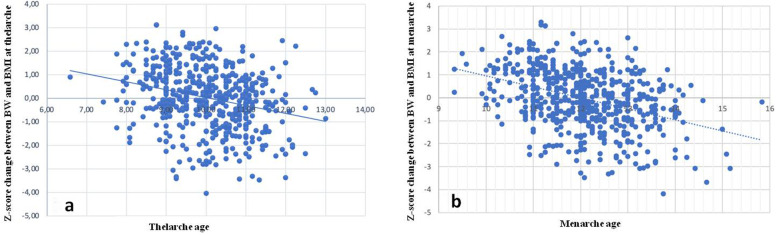
Correlation between age at thelarche (B2) and the Z-score change between BW and BMI at B2. (R: 0.27; *p* < 0.0001; **a**) and between age at menarche and the Z-score change between the previously mentioned parameters (R: 0.38; *p* < 0.0001; **b**)

We found a significant correlation (R: 0.27; *p* < 0.0001) between age at thelarche and the Z-score change between BW and BMI at B2. (Fig. 1a) This correlation is slightly less pronounced than that present between age at thelarche and BMI Z-score at B2 (R: 0.34; *p* < 0.0001) (Table 2).

**Table 2:** Correlation values regarding BMI Z-score at age at thelarche and menarche, the Δ between birth weight Z-score and BMI Z-score at thelarche and menarche and the Δ of BMI Z-score between thelarche and menarche

Weight changes parameters	Auxological parameters	Correlation coefficient (R)	*P* value
BMI Z-score at thelarche	Age at thelarche	0.34	< 0.0001
BMI Z-score at menarche	Age at menarche	0.46	< 0.0001
Δ BW vs. BMI Z-scores at thelarche^a^	Age at thelarche	0.27	< 0.0001
Δ BW vs. BMI Z-scores at menarche^a^	Age at menarche	0.38	< 0.0001
Δ BW vs. BMI Z-scores Δ at menarche^a^	Tempo of puberty	0.20	< 0.0001
Δ BMI vs. BMI Z-scores^b^	Tempo of puberty	0.13	< 0.05
Δ BMI vs. BMI Z-scores^a^	Final height Z-score	0.13	< 0.05

*BW* birth weight, *BMI* body mass index

^a^Birth weight Z-score and BMI Z-score changes; ^a^BMI Z-score changes between thelarche and menarche

We also found a statistically significant relationship between age at menarche and the Z-score change between BW and BMI (R: 0.38; *p* < 0.0001). (Fig. 1b) This correlation is weaker than that between age at menarche and BMI Z-score at the time of the first menstrual cycle (R: 0.46; *p* < 0.0001) (Table 2).

**Fig. 2:**
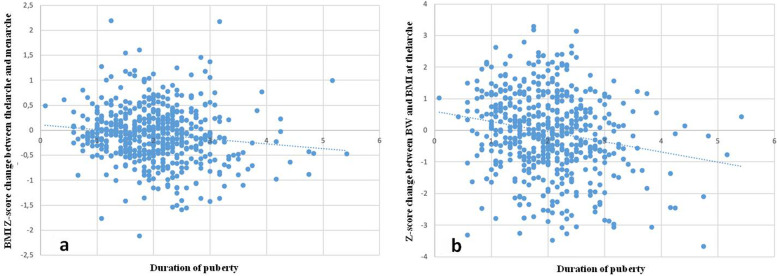
Relationship between the tempo of puberty and the Z-score change between BW and BMI at menarche (R: 0.13; *p* < 0.05; **a**) and between the tempo of puberty and the Z-score change between BW and BMI at B2 (R: 0.20; *p* < 0.0001; **b**)

We also evaluated the relationship between the duration (*tempo*) of puberty and the BMI Z-score change between thelarche and menarche. We observed a statistically significant relationship between these parameters (R: 0.13; *p* < 0.05) (Fig. 2a). This result could be indicative of a further contribution of weight gain after entering puberty in determining the age of onset of menarche and in regulating the end of pubertal growth. We ascertained a statistically valid relationship between the *tempo* of puberty and the Z-score change between BW and BMI at B2 (R: 0.20; *p* < 0.0001) (Fig. 2b) (Table 2) suggesting that weight gain in the first years of life has a significant effect on the timing of puberty (11–13).

We evaluated the influence of BW on the age of onset of pubertal development. There was no statistical correlation between age at B2 and BW Z-score (*p* > 0.05).

Finally, we calculated the average final heights of the girls in our study: 161.51 ± 6.23 cm, in line with the average height of our previous study (2). We did not observe a statistically significant relationship between definitive height and age of appearance of the breast budding. We did, however, observe a linear relationship (R: 0.13; *p* < 0.05) between Z-score change in BMI between thelarche and menarche and definitive height Z-score. The direction of the trend line suggests that a greater variation in weight during puberty (and therefore a shorter duration) is associated with a lower final height of girls.

## Discussion

Our data confirm a clear relationship between BMI and age at thelarche and menarche, as is reported in the literature [2, 5–8]. They also suggest that the Z-score change between BW and BMI is able to influence the timing of puberty and age at menarche, confirming the relationship between earlier pubertal maturation and increased BMI or adiposity in girls [2] and indicating the importance of the early weight gain from birth in the timing of the appearance of these phenomena.

Interestingly, the variation in BMI between age at thelarche and menarche and the Z-score change between BW and BMI at thelarche also has a relationship with the ‘*tempo*’ of puberty, suggesting that early weight gain between BW and BMI at thelarche also influences the duration of puberty. Among the numerous factors that could influence the timing and *tempo* of pubertal development are foetal nutrition, birth weight, childhood dietary habits, physical activity, psychological factors, exposure to electromagnetic fields (EMF) and/or endocrine disrupting, the activating effect of leptin on GnRH—gonadotropin axis and hyperinsulinemia related to obesity [21].

Many data have demonstrated an association between adiposity and early puberty, particularly in girls; for example, a Pediatric Research in Office Settings (PROS) study showed that 6–9-year-old girls with breast development had higher BMI z-scores compared to prepubertal girls [6], and that the prevalence of excess weight is significantly higher in girls with early puberty than in controls [9]. The National Health Examination Survey (NHES 1963–1970) and National Health and Nutrition Examination Survey III (NHANES 1988–1994), examining trends in menarche age over 25 years, reported that age at menarche decreased from 12.7 to 12.5 years whereas the percentage of girls classed as overweight increased from 16 to 27% [22]. Early menarche is more prevalent in overweight girls than in normal weight girls [23], suggesting a causality between increasing obesity and lower age at menarche.

Unfortunately, many of these studies are cross-sectional. Longitudinal studies investigating the association between changes in body composition and pubertal timing and *tempo* in girls are very poor [24], because they relate to only one pubertal parameter [7] or use self-reported data [25].

Our data do not show a significant correlation between age at thelarche and adult height, supporting previous data in the literature [26], although there is a negative relationship between the change in BMI Z-score from thelarche to menarche and adult height. An increase in BMI during puberty is related to a reduced adult height, probably due to the reduced duration of puberty in these girls.

Some data clearly show that higher BMI Z-scores in infancy and childhood are associated with faster length/height velocity in early life, while higher BMI z-scores during mid-childhood are associated with slower length/height velocity during adolescence [27]. This may be particularly important for girls with fast puberty, considering that puberty may follow a nonlinear trajectory with significant interindividual differences of timing and tempo [2, 28]. Further studies will be necessary to better evaluate this finding.

Our study is limited by the fact that we did not have data on weight change for patients with precocious puberty. Future studies would ideally include reliable measurements of body fat mass as well as BMI, hormonal markers for pubertal development and be longitudinal to overcome genetic, nutritional and environmental variables.

In conclusion, our data confirm a significant relationship between BMI and age at B2 and menarche in Italian girls, as well as a clear relationship among weight changes during the first years of life, age at thelarche and menarche and the duration of puberty. There is increasing evidence that environmental factors explain consistent and continuous variations in the onset and *tempo* of puberty.

## Data Availability

Yes
